# Oral Health and Liver Disease: Bidirectional Associations—A Narrative Review

**DOI:** 10.3390/dj10020016

**Published:** 2022-01-21

**Authors:** Fredrik Åberg, Jaana Helenius-Hietala

**Affiliations:** 1 Transplantation and Liver Surgery, Helsinki University Hospital, University of Helsinki, P.O. Box 340, 00029 Helsinki, Finland; 2 Oral and Maxillofacial Diseases, Helsinki University Hospital, University of Helsinki, P.O. Box 281, 00029 Helsinki, Finland; jaana.s.helenius@helsinki.fi

**Keywords:** cirrhosis, oral infections, periodontitis, dysbiosis, oral-gut-liver axis

## Abstract

Several links between chronic liver disease and oral health have been described and are discussed in this narrative review. Oral manifestations such as lichen planus, ulcers, xerostomia, erosion and tongue abnormalities seem to be particularly prevalent among patients with chronic liver disease. These may be causal, coincidental, secondary to therapeutic interventions, or attributable to other factors commonly observed in liver disease patients. In addition, findings from both experimental and epidemiological studies suggest that periodontitis can induce liver injury and contribute to the progression of chronic liver disease through periodontitis-induced systemic inflammation, endotoxemia, and gut dysbiosis with increased intestinal translocation. This has brought forward the hypothesis of an oral-gut-liver axis. Preliminary clinical intervention studies indicate that local periodontal treatments may lead to beneficial liver effects, but more human studies are needed to clarify if treatment of periodontitis truly can halt or reverse progression of liver disease and improve liver-related outcomes.

## 1. Introduction

Associations between oral health and various systemic diseases are well described [1]. Recent studies have also linked oral diseases specifically with liver disease [2,3,4]. Such links can in part be explained by the many shared risk factors between oral disease and liver disease, such as diabetes, smoking and alcohol use [1,2]. Moreover, there may be co-existent diseases; for instance, primary biliary cholangitis (PBC) often co-exists with Sjögren’s syndrome, which in turn is related to oral diseases [5]. In addition to this, periodontitis has more recently also been suggested as a cause or co-factor in the onset and progression of liver disease [2,3,4].

In the late 1800s and early 1900s, seminal work by Dr. Miller, Dr. Billings, Dr. Price, Dr. Rosenow and others described a link between oral diseases and systemic infectious and non-infectious disease [6,7]. An upsurge in this research field occurred in the late 1980s following new reports of an association between poor dental health and acute myocardial infarction [8]. Dental infections, i.e., dental infectious foci, include periodontitis, caries and pericoronitis and if left untreated, may aggravate general health.

Based on the focal infection theory, bacteria, endotoxins (such as lipopolysaccharide, LPS), and locally-produced inflammatory mediators may enter the blood stream from the inflamed periodontal tissues, and thereby cause low-grade chronic systemic inflammation and affect remote organs [1,6]. Especially among immunocompromised patients, it is widely recommended to eradicate all oral and dental infectious foci before major surgeries and medical treatments in order to prevent systemic complications. More recent studies also propose a mechanism where swallowed periodontal bacteria are able to translocate to the gut and cause gut dysbiosis and increased intestinal permeability, and subsequent endotoxemia and inflammation [1,9,10,11]. Interestingly, gut dysbiosis, impaired intestinal barrier, and systemic and liver inflammation, are indeed key mechanisms in the onset and progression of chronic liver disease such as non-alcoholic fatty liver disease (NAFLD) and alcohol-related liver disease (ArLD) [12,13,14,15].

Recent animal studies have demonstrated biologically plausible mechanisms whereby experimental periodontitis can induce liver injury [1,2]. Epidemiological studies have further supported this concept by reporting independent associations between periodontitis and liver disease even after adjusting for multiple confounders [16]. These observations have brought forward the hypothesis of an oral-gut-liver axis [3,17], justifying prospective trials with the intention of influencing liver-related outcomes through periodontal treatments [18,19].

Herein we review the bidirectional associations between oral health and liver disease. The first part of this article discusses the occurrence of various oral manifestations in patients with liver disease, and the second part reviews the evidence underlying the oral-gut-liver axis hypothesis.

## 2. Methods

In this narrative review, we searched PubMed from 1966 onwards using MESH-terms “oral health”, “oral disease”, “periodontitis” and “liver disease”, “cirrhosis”, “NAFLD”, “alcohol”, “liver fibrosis”, “steatosis”, and “steatohepatitis”. We considered all types of peer-reviewed and full-length studies in English.

## 3. Oral Manifestations in Chronic Liver Diseases

Patients with chronic liver disease in general have poor oral health with several oral symptoms and signs (Table 1). These may be causal, coincidental, secondary to therapeutic interventions, or attributable to other factors that patients with liver disease may have in common [20].

### 3.1. Oral Mucosa

Depletion of iron, vitamin B12 or folic acid, which is common in liver disease, may compromise mucosal integrity and predispose to oral mucosal diseases. Since the oral mucosal membrane is very similar to the inner lining of the intestine, similar lesions may be found in the oral cavity and in the other parts of the gastrointestinal tract.

Oral lichen planus (OLP) is a common chronic inflammatory condition that can affect skin and mucous membranes, including the oral mucosa. A strong link exists between hepatitis C virus (HCV) infection and OLP [35]. Oral lichenoid lesions and leukoplakia have also been reported in non-viral chronic liver disease [36]. Early diagnosis by biopsy is essential since small percentage of these lesions may progress to oral squamous cell carcinoma.

Primary sclerosing cholangitis is a rare liver disease, strongly associated with inflammatory bowel disease (IBD). Recurrent aphthous ulcers are painful lesions of the oral mucosa and may appear even several years before the IBD diagnosis [39,40]. Assessment and recognition of these lesions is crucial, and the dentist may be the first clinician to suspect IBD or associated liver disease.

### 3.2. Xerostomia

Xerostomia refers to a subjective feeling of dry mouth, in contrast to objectively measured hyposalivation [45]. Xerostomia is common in liver disease patients and potentially detrimental to oral health and quality of life [26,29,46].

PBC may co-exist with other autoimmune conditions through shared immunogenetic susceptibility. The strongest association is with Sjögren’s syndrome, most frequently, secondary “sicca complex”, although association exists with primary Sjögren’s syndrome as well [5]. The management of symptoms of the sicca complex can be an important part of controlling the overall symptom burden in PBC. 

A fissured tongue is common in dry mouth, and its prevalence in liver disease is around 30–40% [43]. Smooth atrophic tongue is typical in ArLD [42]. Fissured tongue and atrophic tongue were significantly associated with hyposalivation in a study of 300 liver transplant candidates [20].

Systemic diseases and many medications may impair salivary flow [47]. Dry mouth is a major risk factor for dental caries and predisposes to periodontitis and oral mucosal lesions, such as candidiasis, angular cheilitis, and painful stomatitis, all of which have been reported in chronic liver disease [33,41]. 

### 3.3. Teeth

Early childhood liver disease such as biliary atresia can cause discoloration of the developing permanent teeth [23,24]. In addition, increased bilirubin levels in cholestasis can result in greyish-green discoloration of the dentinal layer of the dental hard tissues. Developmental disturbances may also cause enamel hypoplasia, which appear as white or yellowish patches on the teeth. These abnormalities can be corrected by appropriate dental procedures, which can lead to substantial improvement in quality of life [25,48].

Gastroesophageal reflux is common in chronic liver disease and acidic regurgitation may cause tooth erosion when the tooth enamel surface pH falls below 5.5. This can be further aggravated by polypharmacy and consumption of acidic drinks, an observation previously reported in ArLD [33,49].

### 3.4. Periodontal Infections

The prevalence of periodontal disease in patients with liver cirrhosis ranges from 25% to 69% and apical periodontitis from 49% to 79% [31], being considerably more common than in healthy controls [32]. In one study, 63% of liver transplant candidates required tooth extractions prior to transplantation, mainly due to severe caries (deep caries lesions reaching the pulp, root tips, apical periodontitis in non-restorable teeth) to prevent its complications [33]. In that study, poor oral health was associated with PBC, ArLD, and severity of chronic liver disease [33].

A recent study reported that, in patients with periodontitis and liver cirrhosis, there is a unique subgingival microbiota when compared to periodontitis patients without liver disease or healthy controls [30]. The authors hypothesized that periodontitis in liver cirrhosis could be a consequence of dysbiosis secondary to a compromised immune system that renders commensal bacteria pathogenic [30].

Oral infections in liver disease need proper management. There is consensus that all active oral disease needs to be eliminated before liver transplantation in order to prevent infectious complications [50]. A dental treatment plan is made according to the severity of the liver disease with special attention put on preventive measures since nearly all oral diseases are preventable by good oral hygiene.

## 4. Oral-Gut-Liver Axis

### 4.1. Links between Periodontal and Systemic Disease

Periodontal disease is a chronic inflammation of the teeth-supporting tissues that can progress from gingivitis to periodontitis with alveolar bone destruction. If untreated, periodontitis may lead to tooth loss, compromising mastication, aesthetics and quality of life [51].

The oral cavity is the second-largest microbiota reservoir in the body with more than 500 different species identified in adults [52]. Specific bacterial species are enriched in subgingival plaques in advanced periodontitis; these include *Porphyromonas gingivalis* and *Aggregatibacter actinomycetemcomitans* [53].

Globally, periodontitis is a major public health concern with reported prevalence in adults of almost 50% [54], and, in its severe form, ~11% [51].

**Figure 1:**
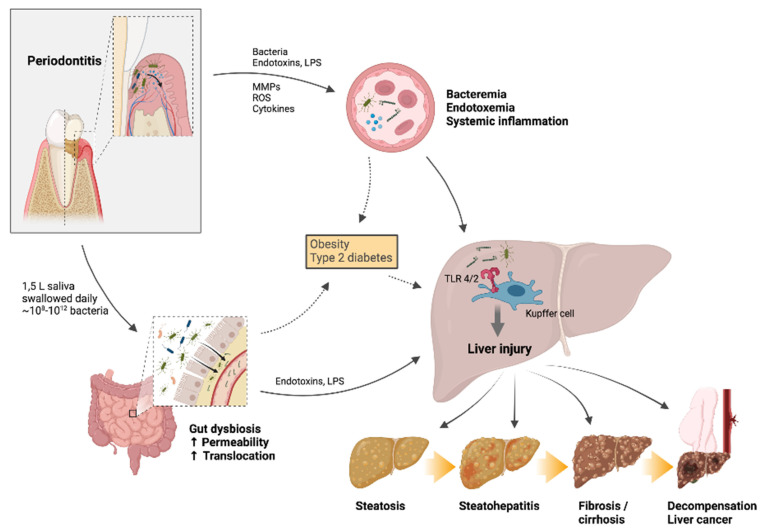
The oral-gut-liver axis hypothesis—possible mechanisms linking periodontitis and liver disease. The oral-gut-liver axis hypothesis—possible mechanisms linking periodontitis and liver disease. Periodontal bacteria, endotoxins (e.g., lipopolysaccharide, LPS), and/or locally produced inflammatory mediators may translocate through the ulcerated epithelium of the periodontal pockets into the circulation, causing bacteremia and systemic inflammation. Alternatively, periodontitis-related bacteria may enter the gut through swallowed saliva and cause gut dysbiosis and impaired intestinal barrier function, thereby resulting in intestinal translocation, endotoxemia and inflammation. These effects may directly or indirectly (through harmful metabolic consequences) damage the liver and contribute to liver steatosis, steatohepatitis, fibrosis and cirrhosis, as well as progression of cirrhosis towards decompensation or liver cancer. Created with BioRender. Abbreviations: LPS, lipopolysaccharide; MMP, matrix metalloproteinase; ROS, reactive oxygen species; TLR, Toll-like receptor.

Periodontal bacteria, bacterial toxins and/or locally-produced or locally-activated inflammation mediators may disseminate to the blood stream and further to extra-oral tissues, where they can cause organ injury (Figure 1) [1]. Experimental animal periodontitis results in increased serum levels of acute phase proteins (e.g., CRP) and inflammatory cytokines [1]. Patients with severe periodontitis exhibit elevated blood levels of endotoxins (e.g., LPS) and pro-inflammatory markers such as IL-1, IL-6 and CRP [1,55].

Daily dental activity (toothbrushing, flossing, chewing), periodontal procedures (e.g., scaling, and root planing) and tooth extractions promote bacteremia [56,57,58,59], and the magnitude of these effects seem to be higher in the presence of periodontitis [60,61].

It is estimated that a person swallows up to 1.5 L of saliva each day, and this can contain 10^8^–10^12^ oral bacteria in the presence of periodontitis [2,9,62]. Despite the acidic gastric environment, the presence of oral bacteria in the gut is not uncommon even among healthy individuals [63], but periodontitis-related bacteria such as *P. gingivalis* (which is acid-resistant) seem to have better capability of colonizing the gut [9]. It seems that swallowed bacteria can, under certain conditions, affect the composition of gut microbiota, resulting in gut dysbiosis, increased gut permeability and translocation, and thereby endotoxemia and systemic inflammation [1,9,10,11]. 

### 4.2. Role of the Gut in Liver Disease

Gut dysbiosis, increased intestinal permeability and translocation, and resultant endotoxemia and inflammation are central pathophysiologic factors in many chronic liver diseases, including NAFLD and ArLD [13,14,64]. All blood from the gut travels via the portal vein to the liver, making the liver constantly exposed to bacterial components and metabolites absorbed from the gut [2]. Endotoxemia and inflammatory mediators can contribute to the development of steatosis, steatohepatitis, fibrosis and cirrhosis [13,14,64,65]. Specifically, pathogen-associated molecular patterns (PAMPs) such as LPS and bacterial RNAs activate pathogen recognition receptors such as the Toll-like receptor 4 (TLR4) on Kupffer cells (liver-resident macrophages) and other immune cells to induce innate immune responses, which contribute to liver disease [13,14].

In a longitudinal general population study, up to 30% of all incident clinical liver disease cases could be attributed to the highest tertile of serum LPS [66], thus supporting the central importance of endotoxemia for population liver disease. Moreover, endotoxemia and systemic inflammation are key drivers of progression of stable liver cirrhosis towards decompensation and acute-on-chronic liver failure [67]. Bacterial toxins can directly cause hepatocyte death and worsening liver function, but also contribute to acute decompensation and organ failure by promoting infections, systemic inflammation and vasodilation [15,67]. In liver cirrhosis, manipulation of the gut microbiota by antibiotics or fecal microbial transplantation leads to beneficial effects [12].

Interestingly, most of the bacterial species enriched in the gut microbiome of liver cirrhosis patients are of oral origin [68]. This suggests a bacterial invasion of the gut from the mouth in liver cirrhosis, possibly facilitated by defective secretion of gastric acid and bile salts in cirrhosis [1]. On the other hand, frequent use of PPIs in patients with liver disease might confound these associations, as PPIs raise gastric pH allowing the presence of bacteria otherwise normally killed by gastric acids [3].

### 4.3. Experimental Periodontitis: Effects on the Liver

Several animal models of periodontitis (reviewed in [4]) have been used for investigating the effects of periodontitis on the liver. In a high-fat diet-induced NAFLD mouse model, intravenous administration of *P. gingivalis* caused significant increases in body and liver weight, serum ALT levels and hepatic triglyceride concentration compared with high-fat diet-induced NAFLD controls [69]. Similarly, odontogenic infection with *P. gingivalis* of mice/rats with NAFLD seems to promote endotoxemia, steatohepatitis, and liver fibrosis through hepatic stellate cell activation [70,71,72,73].

In ligature-induced periodontitis mice models, viable bacteria have been recovered from the liver until the periodontitis-affected teeth were extracted [74].

Several studies have showed that oral administration of periodontitis-related bacteria, including *P. gingivalis* and *Aggregatibacter actinomycetemcomitans*, can lead to gut dysbiosis, decreased gene expression of tight junction proteins in the ileum, and impaired intestinal barrier function, followed by systemic endotoxemia, insulin resistance, and liver steatosis [10,75,76]. In addition, increased amounts of bacteria or bacterial DNA was detected in the liver following such oral administration of periodontopathic bacteria [75,77]. Injection of *P. gingivalis*-derived LPS double-labeled with hydrogen-3 and carbon-14 into the palatal gingiva of rats fed a basal diet or high-fat diet showed that the labeled LPS accumulated markedly in the liver, more so than in other organs [78]. Furthermore, hepatic LPS clearance was delayed in the high-fat diet-fed rats, and LPS contributed to histological liver disease progression [78]. Stimulation of human hepatocellular cells (HepG2) with *P. gingivalis*-LPS seems to promote lipid accumulation and activate proinflammatory pathways [79].

These experimental animal models show that the harmful effects of periodontitis on the liver are exacerbated by pre-existent liver steatosis, with lesser or no liver injury observed in healthy livers [70,76,78,80]. This indicates that periodontitis and NAFLD might synergistically harm the liver, and periodontitis may serve as a co-factor for NAFLD progression. At least one study points to similar synergistic liver injury also between periodontitis and alcohol intake [81].

As one potential explanation for this synergism, studies have shown that liver steatosis and liver cirrhosis are associated with an upregulation of TLR expression, which might sensitize the liver to LPS-induced injury [70,82,83].

In further support of a causal link between periodontitis and liver injury, Tomofuji et al. showed that in a rat model with endotoxemia and liver injury induced by topical application of LPS and proteases, toothbrushing decreased serum LPS levels, liver steatosis and liver inflammation compared to no toothbrushing [84].

### 4.4. Associations between Periodontal Disease and Liver Disease in Human Studies

Several cross-sectional studies have found associations between periodontal disease and elevated liver enzyme levels and liver steatosis [2,4]. Results have varied, however, and in some studies significance of the associations were lost after multivariable adjustment [2]. Nonetheless, two meta-analyses [85,86] reported significant associations between periodontitis and NAFLD, the one by Chen et al. [85] also reported an association with liver cirrhosis.

In the US National Health and Nutrition Examination Survey (NHANES) population sample, adults with moderate-severe periodontitis were more likely to have NAFLD and severe liver fibrosis as evaluated by non-invasive fibrosis tests [87]. This is further supported by a cross-sectional study of patients with biopsy-confirmed NAFLD reporting that periodontitis was significantly more common in patients with NAFLD and steatohepatitis than in NAFLD without steatohepatitis [88]. Furthermore, periodontitis was more common in patients with steatohepatitis and significant fibrosis (F2–4) than in those with steatohepatitis and mild or no fibrosis (F0–1, *p* = 0.04) [88].

In NAFLD patients, serum antibody titers against *P. gingivalis* fimbriae were significantly and independently correlated with histological liver fibrosis stage [72]. In addition, *P. gingivalis* was detected by immunohistochemistry in more than 50% of liver biopsy samples from patients with NASH in another study [70]. 

**Figure 2:**
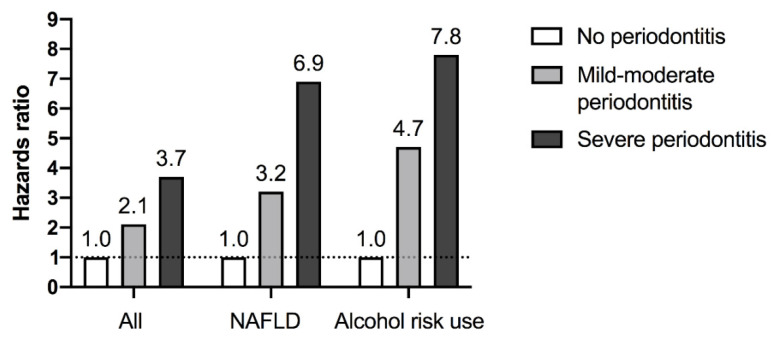
Periodontitis as a risk factor for incident severe liver disease (hospitalization, cancer, death) in a Finnish general population health-examination survey cohort of 6165 men and women [16]. The risk estimates (hazards ratios) were higher among individuals with baseline non-alcoholic fatty liver disease (NAFLD) or alcohol risk use. Abbreviations: NAFLD, non-alcoholic fatty liver disease.

In longitudinal studies, periodontitis have been associated with a rise over time in ALT levels (but not AST) [89] and incident NAFLD [90]. Furthermore, in a Finnish general population health-examination survey cohort of 6165 well-characterized men and women with linked healthcare register follow-up data, we found that mild-moderate periodontitis was associated with a 2-fold increased risk for incident severe liver disease defined as hospitalization, cancer or death related to liver disease, while severe periodontitis was associated with a 3.3–3.7-fold risk after multiple confounder adjustments (Figure 2) [16]. The impact of periodontitis was amplified among subjects with baseline NAFLD or alcohol risk use (Figure 2), which is well in agreement with the aforementioned animal experiments indicating that periodontitis may be particularly relevant in the context of pre-existing susceptibility (liver steatosis).

Liver cancer is an important complication of chronic liver disease. Hepatocellular carcinoma (HCC) constitutes 80% of all primary liver cancers, and most cases develop on a background of pre-existing liver cirrhosis. Two independent cohort studies (UK Biobank, n = 475,766, and the Health Professionals Follow-up study, n = 19,933 men) found that self-reported poor oral health/periodontal disease was significantly associated with incident HCC when adjusted for confounders [91,92]. A similar association was not observed for other gastrointestinal cancers [91]. Corroborating these findings, two other longitudinal cohort studies have reported an association between tooth loss (a crude surrogate of oral health status) and incident liver cancer [93,94]. Given that HCC usually requires the pre-existence of cirrhosis or advanced liver fibrosis, it remains unclear whether these associations represent links to cirrhosis/fibrosis or specifically to HCC. Tamaki et al. observed that patients with HCC and periodontitis tended to have more advanced HCC at diagnosis compared to periodontally healthy HCC patients [95].

In an observational study of liver cirrhosis patients, poor oral health correlated with the progression of liver cirrhosis during the year preceding the dental examination [96]. Other researchers reported that periapical radiolucency as a sign of periapical inflammation was associated with the prevalence of cirrhosis-related complications such as ascites, hepatic encephalopathy and/or variceal bleeding [97], and the presence of dental calculus predicted worse survival among liver transplant candidates [98]. Furthermore, Bajaj et al. found higher endotoxin levels in saliva of cirrhosis patients compared to healthy controls [99], and salivary dysbiosis predicted incident cirrhosis decompensation events [99].

Periodontal disease can exacerbate various metabolic abnormalities such as diabetes and obesity [2], which are also well-known risk factors for liver disease. This may also be an important mechanism linking periodontitis and liver disease.

Additional mechanisms not yet extensively explored in the context of liver disease have also been suggested. Specifically, abundant circulating antibodies against oral bacteria might promote systemic disease through molecular mimicry [100], and large amounts of swallowed dead bacteria might stimulate gut pathogens (necrotrophy and necrovirulence) [9].

### 4.5. Systemic and Liver Effects of Periodontal Treatment

According to a 2014 meta-analysis of interventional trials, treatment of periodontitis generally leads to significant decreases in serum levels of CRP, IL-6, TNF-α, fibrinogen, total cholesterol, and a significant rise in HDL-cholesterol [101]. In adults without systemic disease who had periodontitis and elevated serum LPS levels at baseline, LPS levels significantly decreased by 3 months following periodontal treatment [102]. More recent studies report decreases also in serum levels of reactive oxygen species [103,104] following periodontal treatment. However, a clear reduction in systemic inflammation parameters has not been observed in all studies [105].

A 12-month randomized, controlled trial in patients with type 2 diabetes and moderate-severe periodontitis found that intensive periodontal therapy compared with supra-gingival scaling and polishing resulted in a decrease in HbA1c of 0.6% (95% CI 0.3–0.9; *p* < 0.0001) [106], an effect comparable to that of single antidiabetic drug therapies. Studies with longer follow-up are needed to confirm a sustained benefit of periodontal therapy.

Few human studies have assessed the effect of periodontal treatment on liver disease. In a study of 10 patients with NAFLD and *P. gingivalis*-associated periodontitis, serum levels of ALT and AST decreased significantly at 1 month following periodontal treatment (oral hygiene instruction, scaling and root planing, and local administration of hydrochloric minocycline), with stable or further decreasing transaminase levels seen at 3 months [69]. Body weight remained unchanged during the follow-up. However, there was no control group in that study.

**Figure 3:**
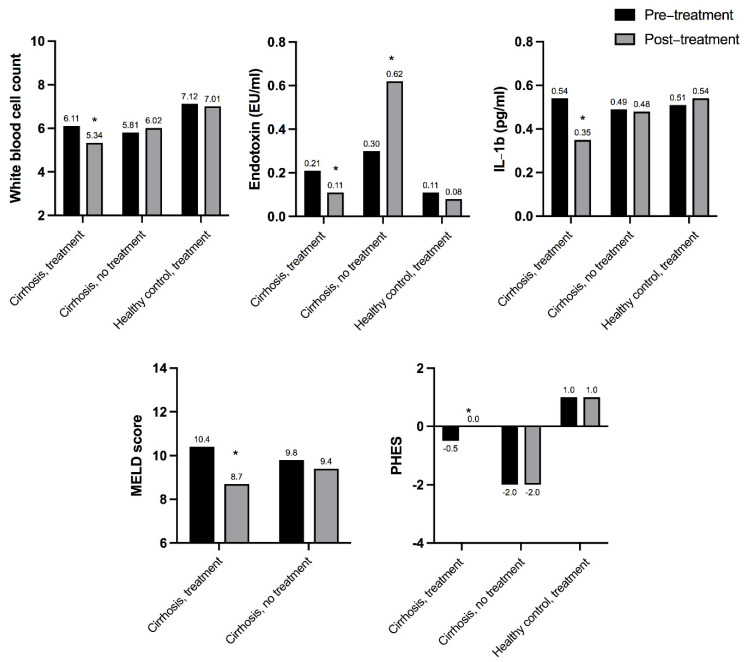
Selected systemic and liver-related effects of periodontal treatment in patients with liver cirrhosis, as compared to cirrhosis patients without periodontal treatment and healthy controls undergoing periodontal treatment in the interventional study by Bajaj et al. [18]. A higher PHES score indicates better cognitive function. * indicates a significant (*p* < 0.05) change from pre- to post-treatment.

Bajaj et al. published a proof-of-concept interventional study comprising 26 liver cirrhosis patients with chronic gingivitis and/or mild-moderate periodontitis [18]. Comparison was to 20 healthy controls with periodontal therapy, and to 24 cirrhosis patients observed without therapy. This was not a randomized trial, but the cirrhosis controls fulfilled the same inclusion and exclusion criteria, and were fairly similar on key baseline characteristics. Patients with abscesses, severe periodontitis or severe cavitated lesions were excluded. Periodontal therapy consisted of prophylaxis or scaling and root planing followed by oral hygiene instructions. Therapy effect was evaluated at 30 days. Following periodontal treatment in cirrhosis patients, the authors observed a reduction in systemic endotoxemia and inflammation, as well as improvements in the MELD score and hepatic encephalopathy test scores (Figure 3) [18]. In addition, favorable changes were seen in the stool microbial composition with increases in autochtonous taxa and decreases in potentially pathogenic and oral-origin taxa. Such microbial changes were seen among both healthy controls and cirrhosis patients, and the changes were more pronounced in those with hepatic encephalopathy at baseline. In contrast, among cirrhosis controls, no changes in either endotoxemia, inflammation or stool microbiota occurred.

Interestingly, among cirrhosis patients with prior hepatic encephalopathy, periodontal treatment not only reduced systemic endotoxemia, inflammation, and gut dysbiosis, it also improved cognition and health-related quality of life despite patients already being on full gut-modulator treatment (lactulose and rifaximin) [18].

Future trials are needed before conclusions can be made on the real clinical impact of periodontal treatment on the course of chronic liver disease. 

## 5. Conclusions

Patients with liver disease often have poor oral health, and special attention is needed in planning their dental treatment. As liver disease progresses, it may aggravate oral health. Regular professional follow-up and special guidance in oral self-care are needed, considering also the potential for malignant transformation of some oral lesions. Close co-operation between the hepatologist and dentist is recommended. The possible impact of good control of the underlying liver disease on oral health needs to be explored in future studies.

The oral-liver link does not seem to simply be a consequence of common and shared risk factors, but seems to be driven, to a substantial degree, by oral infection-induced liver injury. However, more studies in humans are needed, as most of the evidence in humans come from observational studies. Considering that liver cirrhosis is associated with a multidimensional immunologic impairment with a global mucosal-immune change, it is possible that cirrhosis and periodontitis could aggravate each other in a vicious circle. 

Although clinical intervention studies have shown that local periodontal treatments decrease systemic inflammation and endotoxemia, improve metabolic control, and appear to be beneficial in liver disease, clear evidence that the treatment of periodontitis can halt or reverse the progression of liver disease and improve outcome is still lacking. Nonetheless, based on the available evidence, oral examination education regarding oral self-care seems appropriate for all patients with liver disease.

## Figures and Tables

**Table 1:** Oral manifestations commonly seen in patients with liver disease.

Oral Manifestation	Relation to Liver Disease	Reference
Petechiae, telangiectasia, hematoma, gingival bleeding, reduced wound healing	Coagulopathy	de Oliveira Rech 2021 [21] Helenius-Hietala 2016 [22]
Discolorations of teeth, enamel hypoplasia, delayed eruption of teeth	Biliary atresia, malnutrition	Sommer 2010 [23] Olczak-Kowalczyk 2012 [24] Hosey 1995 [25]
Xerostomia Hyposalivation	HCV, PBC, IBD/PSC	Guggenheimer 2003 [26] Lins 2011 [27] Ebert 2012 [28] Helenius-Hietala 2013 [29]
Periodontal disease	Cytopenia, dysbiosis, compromised immune system	Kuraji 2021 [2] Acharya 2017 [3] Jensen 2018 [30]
Tooth decay (caries)	Hyposalivation/xerostomia, alcohol-related liver disease	Gronkjaer 2015 [31] Lins 2011 [27] Silva Santos 2012 [32] Helenius-Hietala 2012 [33]
Erosion	Alcohol-related liver disease, gastric reflux	Dukic 2010 [34] Helenius-Hietala 2012 [33]
Oral lichen planus, lichenoid lesions	HCV, PBC	Lodi 2010 [35] Scully 2008 [36] Helenius-Hietala 2014 [37]
Leukoplakia	HCV	Grossman 2009 [38]
Mucosal ulcers	IBD/PSC, PBC	Elahi 2012 [39] Zbar 2012 [40]
Candidiasis, angular cheilitis	Compromised immune system	Nagao 2012 [41] Helenius-Hietala 2012 [33] Helenius-Hietala 2014 [37]
Glossitis, atrophic tongue	Alcohol-related liver disease, nutritional deficiencies	Cunha 2012 [42]
Fissured tongue	HCV, IBD/PSC	Elahi 2012 [39] Diaz-Ortiz 2005 [43] Helenius-Hietala 2012 [33] Guggenheimer 2010 [20]
Parotid gland enlargement/ sialadenitis	Alcohol-related liver disease, HCV	Guggenheimer 2009 [44]

Abbreviations: HCV, hepatitis C virus; IBD, inflammatory bowel disease; PBC, primary biliary cholangitis; PSC, primary sclerosing cholangitis.

## Data Availability

Not applicable.
